# Multi-Parametric Representation of Voxel-Based Quantitative Magnetic Resonance Imaging

**DOI:** 10.1371/journal.pone.0111688

**Published:** 2014-11-13

**Authors:** Maria Engström, Jan B. M. Warntjes, Anders Tisell, Anne-Marie Landtblom, Peter Lundberg

**Affiliations:** 1 Division of Radiology, Department of Medical and Health Sciences, Linköping University, Linköping, Sweden; 2 Center for Medical Image Science and Visualization (CMIV), Linköping University, Linköping, Sweden; 3 Division of Cardiovascular Medicine, Department of Medical and Health Sciences, Linköping University, Linköping, Sweden; 4 SyntheticMR AB, Linköping, Sweden; 5 Division of Radiation Physics, Department of Medical and Health Sciences, Linköping University, Linköping, Sweden; 6 Division of Neurology, Department of Clinical and Experimental Medicine, Linköping University, Linköping, Sweden; Charité University Medicine Berlin, Germany

## Abstract

The aim of the study was to explore the possibilities of multi-parametric representations of voxel-wise quantitative MRI data to objectively discriminate pathological cerebral tissue in patients with brain disorders. For this purpose, we recruited 19 patients with Multiple Sclerosis (MS) as benchmark samples and 19 age and gender matched healthy subjects as a reference group. The subjects were examined using quantitative Magnetic Resonance Imaging (MRI) measuring the tissue structure parameters: relaxation rates, R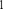 and R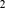, and proton density. The resulting parameter images were normalized to a standard template. Tissue structure in MS patients was assessed by voxel-wise comparisons with the reference group and with correlation to a clinical measure, the Expanded Disability Status Scale (EDSS). The results were visualized by conventional geometric representations and also by multi-parametric representations. Data showed that MS patients had lower R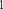 and R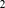, and higher proton density in periventricular white matter and in wide-spread areas encompassing central and sub-cortical white matter structures. MS-related tissue abnormality was highlighted in posterior white matter whereas EDSS correlation appeared especially in the frontal cortex. The multi-parameter representation highlighted disease-specific features. In conclusion, the proposed method has the potential to visualize both high-probability focal anomalies and diffuse tissue changes. Results from voxel-based statistical analysis, as exemplified in the present work, may guide radiologists where in the image to inspect for signs of disease. Future clinical studies must validate the usability of the method in clinical practice.

## Introduction

Magnetic resonance imaging (MRI) is frequently used for diagnosis of brain disorders, such as stroke, brain tumors, and multiple sclerosis (MS). Conventional clinical MRI is generally a qualitative method. This means that eventual tissue pathologies are detected as visible differences in image intensity between pathological and normal tissue. Diffuse pathologies can be particularly difficult to detect since there are no clear contrasting borders between pathological and normal tissue.

In recent years there has been an increasing interest in developing methods for quantitative MRI (qMRI), which provides information about structural differences in brain tissue [1]. Various methods for quantitative measurements of the tissue parameters, such as longitudinal relaxation time (T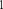), transversal relaxation time (T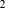) and/or proton density (PD); have been reported in the literature [2]–[10]. Previously, we reported a method for quick, simultaneous measurements of T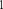, T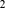, and PD, which also was optimized for clinical usage [11], [12]. This method provides the possibility to compare objective measures of tissue structure within subjects in longitudinal studies and between subjects in comparative studies. As T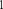, T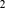, and PD are quantified in each image voxel, the qMRI method allows for voxel-based statistical comparisons within and between subjects. In a recent study, we showed that qMRI together with brain normalization to a standard template could be used to generate reference tissue maps of typical brain characteristics in healthy subjects [13]. In the present study we aimed to explore the feasibility of voxel-based qMRI to *identify T* 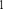 *, T* 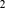 *, and PD tissue properties in these groups*. We aimed to investigate if voxel-based statistical analysis of qMRI data can reveal features in tissue structure that are typical for certain pathologies. In some disease conditions there are discrepancies between radiological and clinical measures. A well-known example is the clinico-radiological paradox in MS [14]. A second aim was therefore to investigate if correlation between normalized qMRI data and *clinical measures* can provide additional information about anatomical location of the pathologies that are related to specific symptoms in a patient group.

MR images are most often represented using a geometric representation in anatomical space where eventual lesions are related to certain anatomical structures. The qMRI method provides alternative opportunities to represent data, which are only little investigated. Here, we explore the possibilities of *multi-parametric visualization* for the ability to provide information about trends in the development of brain pathologies, reflected by objective measures in a patient group. As many neurological disorders are caused by focal rather than global pathologies, we also aimed to investigate if analyses in *regions of interest* (ROIs) using a generally accessible, standard brain atlas can provide additional information about local pathological changes.

We hypothesized that voxel-based qMRI and multi-parametric representation could be used to detect disease-specific pathologies, for example ‘lesion probability’ and ‘diffuse tissue’ changes that are difficult to detect by conventional neuroimaging methods. In order to demonstrate the feasibility of the voxel-based qMRI method, we selected a small group of healthy individuals and a group of MS patients as benchmark samples. The reason for choosing MS as benchmark is that this disease is characterized both by discrete lesions with high lesion frequency in certain anatomical structures and diffuse, globally spread white matter changes [3].

This work is a continuation of previously published works on methods for fast qMRI acquisition [11], [12] and voxel-based analyses in a healthy reference group [13]. The overall aim with the present work was to further explore the opportunities of voxel-based analysis and multi-parametric representations of qMRI data from two different groups. Here we show that qMRI can be used for differentiation of tissue properties in a group of MS patients, and that multi-parametric representations provide additional information compared to conventional geometric representations in anatomical space.

## Results

### Group-level Tissue Characterization

**Figure 1:**
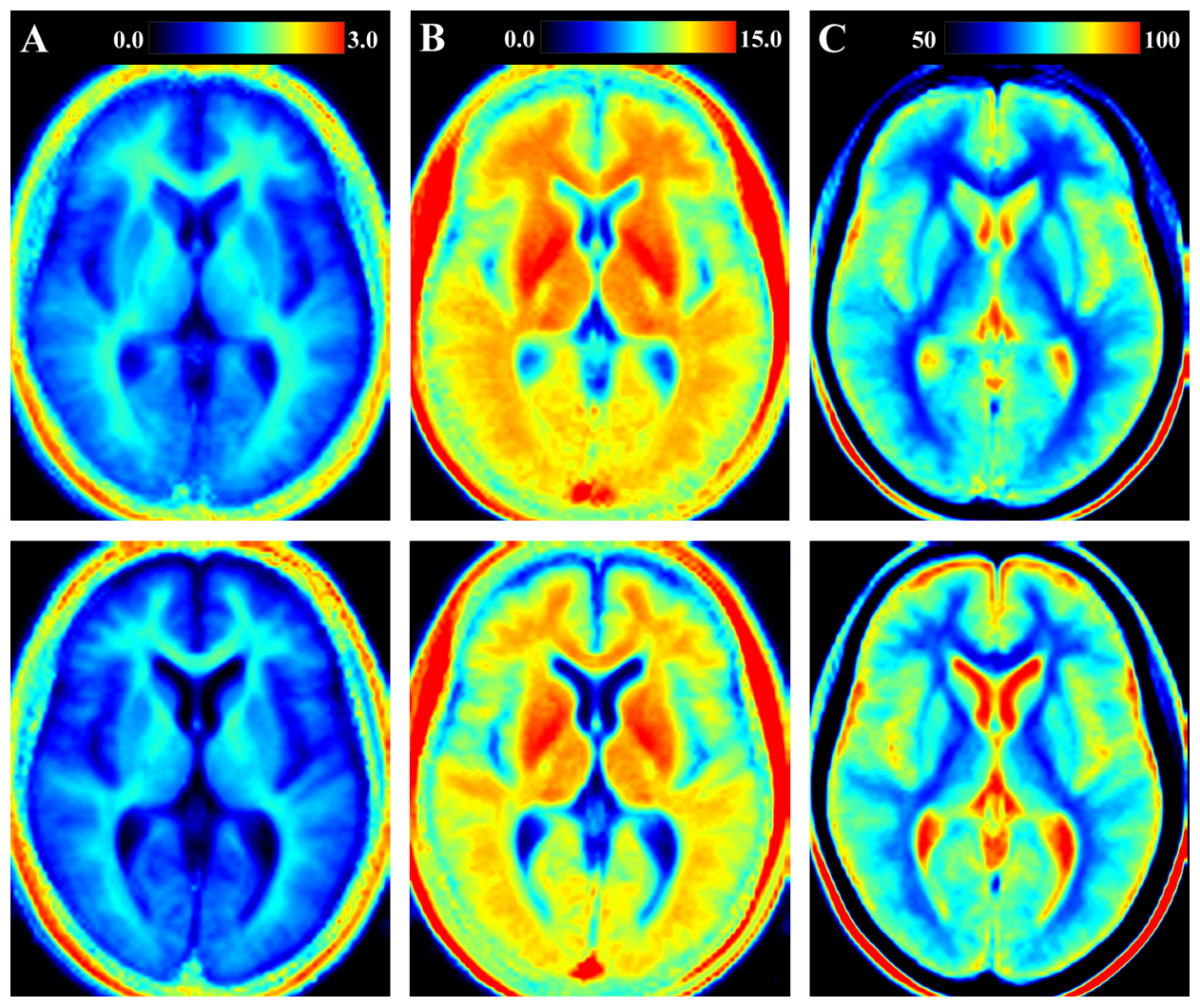
Geometrical representation of quantitative tissue parameters. The figure shows a selected slice of the quantitative tissue maps in the reference group of healthy subjects (top row) and the group of multiple sclerosis (MS) patients (bottom row). A) longitudinal relaxation rate (R_1_) on a scale 0–3 s^−1^; B) transversal relaxation rate (R_2_) on a scale 0–15 s^−1^; C) proton density (PD) on a scale 50–100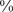, where 100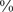 corresponds to pure water at 37°C.

**Figure 2:**
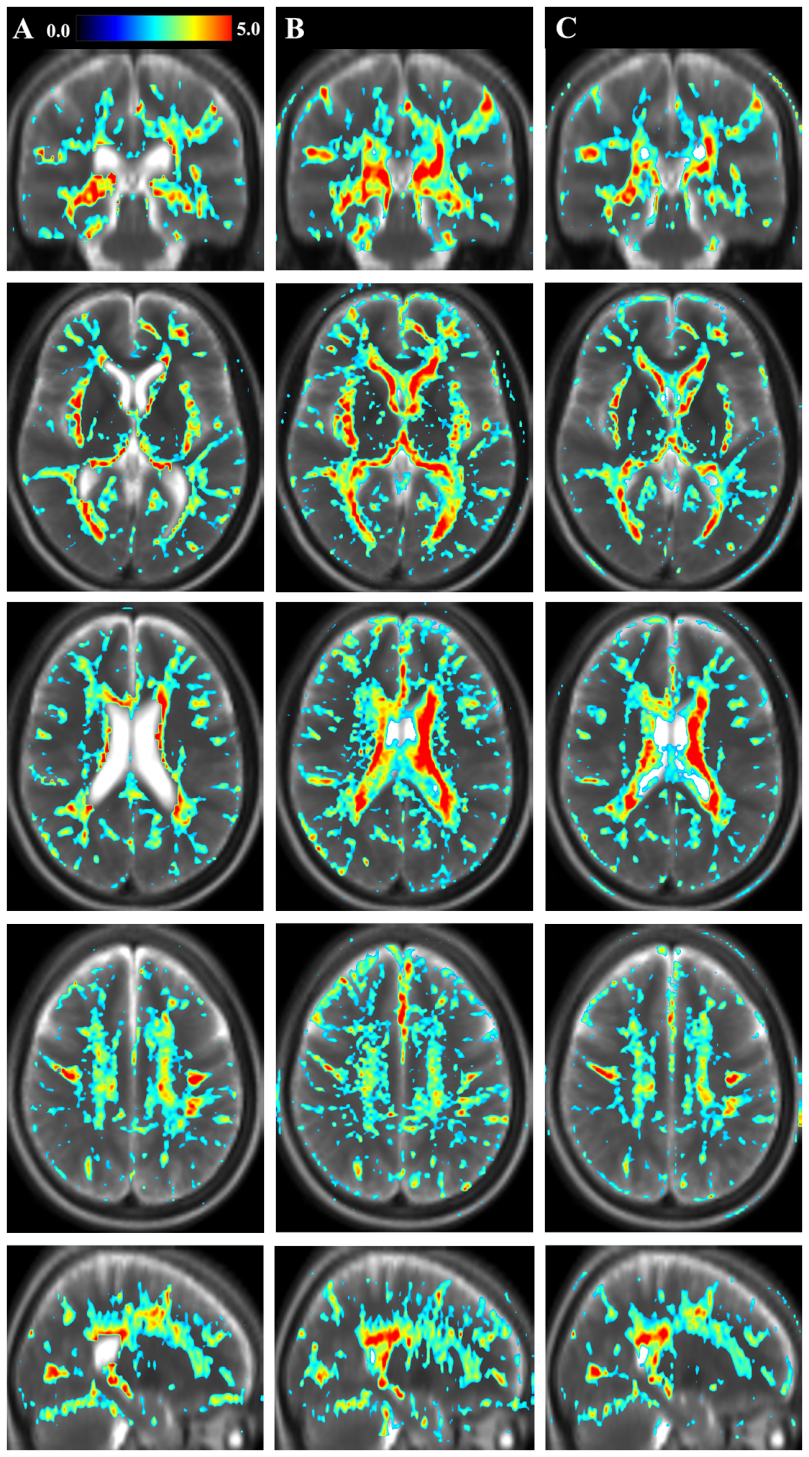
Geometrical representation of tissue parameter differences between groups. The figure shows differences in tissue parameters between the group of Multiple Sclerosis (MS) patients and the reference group. Shown as the color overlay is the T-statistics of the voxel-based differences between groups. A) longitudinal relaxation rate (R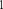); B) transversal relaxation rate (R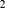); C) proton density (PD) in one coronal, three axial and one sagittal slice. The color scale represents T  = 0.0–5.0. The figure shows uncorrected statistics, thresholded at T = 2. In the background a synthetic T2-weighted image of the same slice is displayed for visual guidance.

In Figure 1 the averaged, normalized R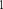, R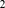, and PD maps are shown for a single slice in the reference brain (top row) and the MS benchmark brain (bottom row). It is clearly seen that cerebrospinal fluid (CSF), WM, and GM have different characteristics and the maps can discriminate between the different tissue types using any of the three parameters. By inspecting the images it is, however, not completely evident to discriminate differences in tissue characteristics between the two groups. By voxel-based statistical analysis, on the other hand, distinct tissue differences between MS patients and the reference group were detected very clearly. In general, MS patients had lower R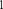 and R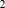, and higher PD as compared to the reference group. Figure 2 shows differences in R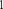, R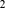, and PD between MS patients and the healthy reference group using a statistical threshold of T = 2. By inspecting Figure 2, marked differences between MS patients and healthy subjects are shown in periventricular WM and in wide-spread areas encompassing especially central and sub-cortical WM structures. In peripheral brain, only a few structures with differences between the groups appeared, but a number of sulci, mainly in the frontal and parietal lobes were highlighted. Correcting for multiple comparisons, we observed significant differences, p
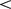
0.05, when testing for R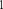 and R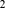 lower in MS, and PD higher in MS in all regions, except occipital white matter when testing for PD (p = 0.07). No significant results were observed for the reverse comparisons. The analysis of the three different tissue structure parameters, R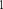, R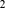, and PD, yielded similar, but not identical results, which will be discussed further below.

The intracranial volume was similar in both groups: 1400
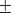
131 mL and 1357
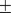
95 mL in the reference group and in MS patients, respectively. The difference in intracranial volume between the groups ( = 43 mL) was not significant (p = 0.3). The brain parenchymal fraction, however, was 89.9
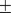
2.4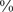 for the reference group and 82.1
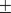
4.5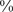 for the MS group: a significant difference of 7.7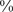 (p
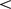
0.0001). The ventricular fraction was 1.0
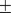
0.5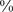 for the reference group and 2.5
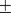
1.2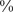 for the MS group: a significant difference of 1.5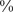 (p
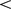
0.0001).

### Relation to Clinical Measures

**Figure 3:**
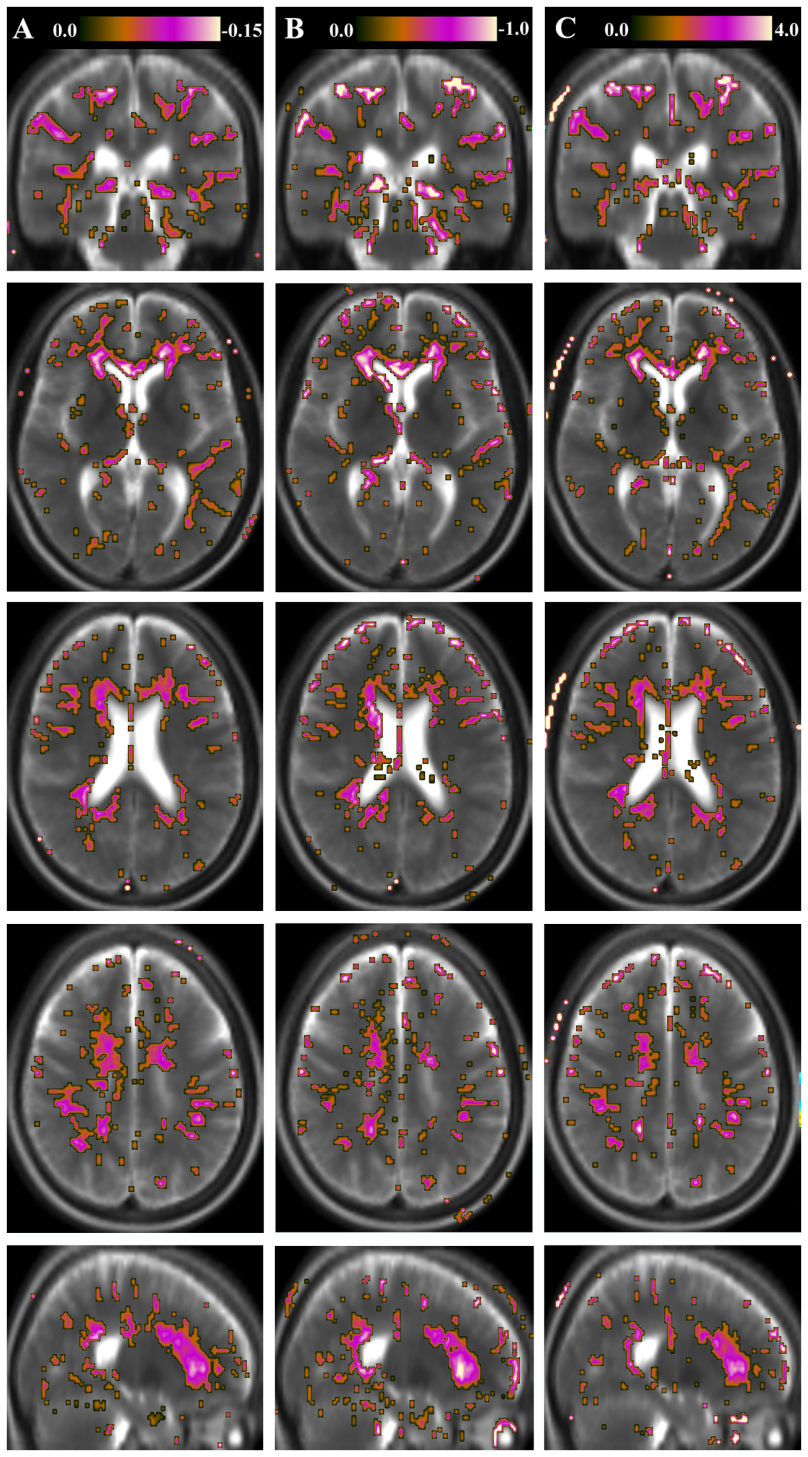
Geometrical representation of the correlation of tissue parameters and clinical measures. The figure shows the geometrical representation of the correlation of R
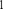
–R
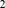
–PD values with a clinical measure for the group of Multiple Sclerosis (MS) patients. Shown as the color overlay is the slope of the voxel-based correlation with Expanded Disability Status Scale (EDSS). A) longitudinal relaxation rate (R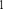) on a scale r = 0.0 to -0.15; B) transversal relaxation rate (R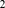) on a scale r = 0.0 to -1; C) proton density (PD) on a scale r = 0.0–4.0. The correlation images are shown in the same slices as in Figure 2. The figure shows uncorrected statistics, thresholded at T = 2. In the background synthetic T2-weighted images of the MS group in the same slices are displayed for visual guidance.

In Figure 3, the geometrical representations of the correlation between tissue parameters and a clinical measure, the Expanded Disability Status Scale (EDSS), are shown. When comparing the EDSS correlation maps with the results in Figure 2, which shows voxel-based differences between MS patients and the reference group, some overlap was observed, especially in periventricular WM and the corona radiata. However, there were also features with distinct differences between the two representations. Notably, MS-related tissue pathology in frontal WM in, and adjacent to, the corpus callosum was highly correlated with EDSS, while WM changes in the posterior corpus callosum were highlighted in the group difference representations. These features are most clearly visualized in the sagittal images in Figures 2 and 3, which show uncorrected results. In this study, the differences between MS patients and the reference group were significant when correcting for multiple comparisons, whereas the EDSS correlations were not significant at the corrected level.

The partial overlap between the results of Figure 2 and Figure 3 suggests a weak correlation between group differences (Figure 2) and the MS-EDSS relation (Figure 3). To confirm this, we performed a linear regression of the T-maps presented in Figure 2 and the slope data presented in Figure 3. All data were included in the analysis, also non-significant data not plotted in Figures 2 and 3. The goodness of fit R^2^ of the regression was 0.50 for R_1_, 0.48 for R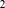, and 0.46 for PD. The observed slopes were 0.53, 0.49, and 0.50 for R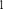, R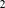, and PD, respectively.

### Multi-parametric visualization

**Figure 4:**
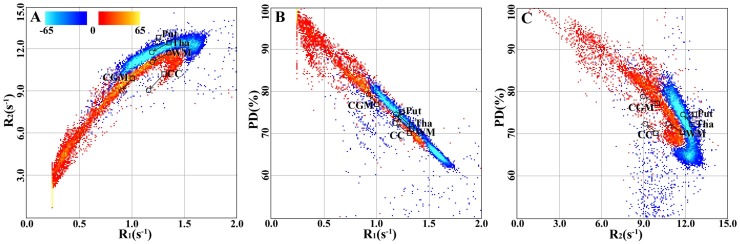
Multi-parametrical representation of tissue parameters in groups. The figure shows whole-brain differences in R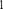-R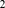-PD values between the group of Multiple Sclerosis (MS) patients and the reference group. A) R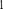-R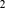 values; B) R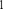-PD values; C) R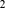-PD values. The color scales indicate number of voxels. Blue color indicates a larger number of voxels in the reference group. Red color indicates a larger number of voxels in the MS group. The markers indicate the positions of the mean values in cortical grey matter (CGM), thalamus (Tha), putamen (Put), white matter (WM), and corpus callosum (CC). The mean values are taken from the decreased ROIs presented in Table 2. A square is used for the reference group and a circle for the MS group. The arrows point in the direction from the reference group to the MS group.

In Figure 4 the multi-parametric representations of tissue parameters of the whole brain are projected as 2-dimensional graphs for each R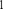-R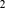, R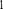-PD, and R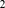-PD pair. The differences between the reference group and the MS group are visualized in two colors in the multi-parametric representations. The blue color scale indicates a larger number of voxels with a specific parametric location in the reference group compared to the MS group. Correspondingly, the red scale indicates a larger number of voxels in the MS group. Only voxels with signal intensity higher than 10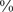 of the maximum signal intensity (*e.g.* 395451 out of 510340 voxels) were taken into account. In this way, differences between the groups are visualized in the multi-parametric space and indications of the direction of disease-specific tissue changes are provided. In Figure 4A, it can be seen that the direction of change due to MS is towards lower R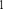 and R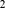 values. In Figures 4B and 4C the MS-specific changes point towards higher PD values.

**Figure 5:**
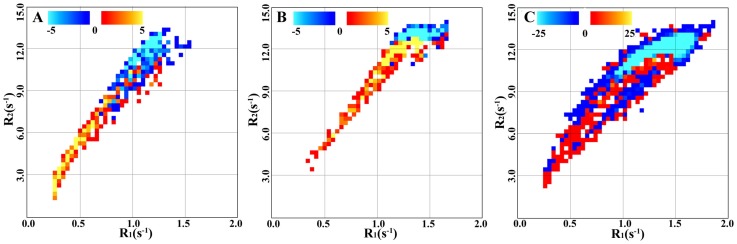
Multi-parametrical representation of tissue parameters in regions of interest (ROIs). The figure shows differences in R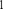 and R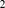 values between Multiple Sclerosis (MS) patients and the reference group for three separate cerebral ROIs. A) Caudate nucleus; B) Thalamus; C) Total white matter. The ROIs were all reduced with 2 mm to avoid partial volume effects at the edges. Only the R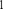-R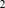 projections of the R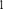-R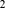-PD space are shown. The color scales indicate number of voxels. Blue color indicates a larger number of voxels in the reference group. Red color indicates a larger number of voxels in the MS group.

In Figure 5, the parametric representation in 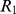
–R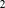 space is visualized for three selected ROIs: the caudate nucleus, the thalamus, and the total WM. Each ROI was reduced with 2 mm in comparison to the ROIs in the standard atlas to avoid large influence of partial volume at the ROI edges. Since the multi-parametric representations in Figure 5 contained a far lower number of included voxels compared to the representations in Figure 4, the number of bins was chosen to 50 rather than 200 as in Figure 4.

### Tissue properties in regions of interest

Figure 2 shows that the MS patients in general had lower R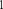 and R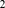, and higher PD compared to the reference group. In Table 1 and 2 it is seen that this general feature also apply to all investigated ROIs. The difference in tissue parameters between the reference group and MS patients were highly significant, p
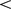
0.001. For the reduced ROIs (Table 2), we observed two tendencies: in some ROIs the magnitudes of the parametric values increased whereas in some ROIs the values decreased. For example, R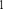 and R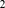 increased and PD decreased with ROI reduction in the ventricles, whereas the opposite trend was observed for the corpus callosum and the pons.

**Table 1:** Descriptive data from the region of interest (ROI) analysis using standard templates.

	ROI size	Reference group			Multiple Sclerosis group		
Structure	(voxels)	R 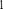 (s 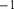 )	R 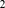 (s 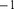 )	PD (%)	R 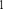 (s 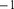 )	R 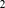 (s 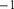 )	PD (%)
Lateral ventricles	2349	0.72 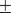 0.56	6.6 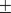 4.2	85 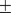 14	0.40 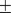 0.47	4.1 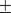 3.7	92 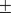 12
Insula	3628	0.94 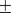 0.35	9.8 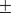 2.7	80 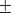 9	0.87 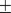 0.39	9.0 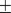 3.1	81 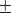 10
Cingulate cortex	7655	1.09 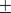 0.36	10.7 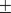 2.2	77 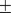 9	0.96 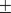 0.39	9.7 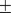 2.8	80 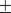 10
Caudate nucleus	1956	1.08 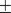 0.43	10.5 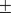 3.4	78 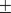 10	0.75 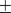 0.51	7.4 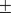 4.5	85 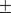 11
Cortical gray matter	53401	1.02 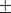 0.57	10.0 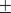 3.2	75 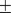 20	0.92 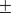 0.61	9.1 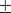 3.5	77 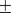 20
Pons	2518	1.06 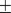 0.43	9.8 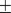 3.5	76 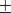 11	0.94 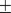 0.50	8.6 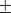 4.2	78 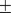 14
Putamen	2073	1.29 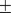 0.18	12.9 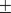 1.2	74 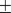 5	1.25 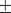 0.21	12.4 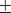 1.5	74 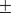 6
Mid brain	2289	1.18 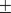 0.36	10.8 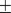 3.0	74 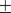 8	1.10 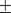 0.39	10.1 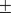 3.4	76 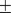 9
Thalamus	2157	1.29 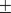 0.25	12.1 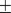 1.9	73 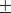 7	1.12 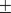 0.41	10.6 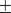 3.5	76 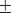 10
Occipital white matter	11077	1.22 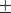 0.36	11.6 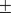 1.9	74 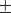 9	1.13 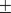 0.34	11.1 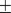 2.2	76 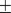 9
Frontal white matter	40008	1.17 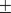 0.46	10.8 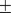 2.7	74 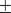 14	1.05 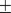 0.48	9.9 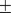 3.1	77 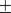 14
Parietal white matter	14362	1.18 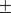 0.40	11.0 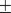 2.5	74 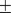 11	1.06 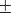 0.42	10.2 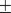 3.0	77 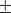 12
Sublobar white matter	12855	1.25 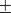 0.41	10.9 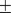 2.8	73 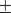 10	1.09 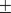 0.48	9.6 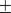 3.6	76 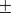 12
White matter	106935	1.20 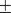 0.41	11.1 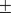 2.4	74 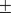 12	1.07 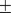 0.43	10.2 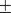 2.9	77 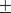 12
Corpus callosum	2685	1.20 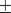 0.55	9.7 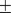 3.7	73 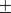 13	0.99 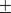 0.59	8.1 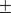 4.1	77 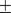 14

The table shows ROI sizes, mean R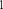, R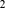, and proton density (PD) values, and standard deviations of 15 pre-defined structures in the Montreal Neurological Institute (MNI) standard brain template. All comparisons between the reference group and the multiple sclerosis group were highly significant, p
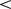
0.001.

**Table 2:** Descriptive data from the region of interest (ROI) analysis using cropped templates.

	ROI size	Reference group			Multiple Sclerosis group		
Structure	(voxels)	R 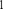 (s 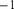 )	R 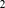 (s 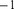 )	PD (%)	R 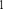 (s 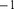 )	R 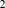 (s 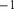 )	PD (%)
Lateral ventricles	96	0.29 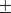 0.33	3.4 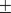 2.9	96 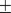 9	0.20 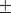 0.17	1.7 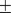 1.4	99 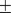 5
Insula	1634	0.82 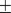 0.30	9.0 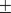 2.7	83 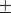 8	0.73 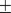 0.35	8.0 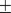 3.2	84 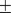 9
Cingulate cortex	7140	1.00 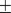 0.32	10.4 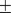 2.1	79 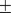 8	0.87 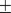 0.35	9.3 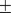 2.8	82 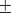 10
Caudate nucleus	1000	1.03 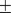 0.36	10.6 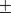 3.1	80 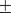 8	0.64 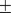 0.45	6.9 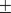 4.4	87 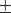 10
Cortical gray matter	306 1	.01 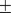 0.53	10.0 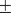 3.1	77 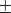 17	0.89 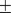 0.53	8.9 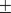 3.5	79 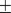 17
Pons	2654	1.06 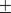 0.43	9.8 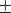 3.5	76 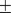 11	0.94 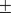 0.50	8.6 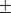 4.2	78 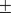 14
Putamen	1252	1.29 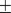 0.18	12.9 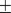 1.2	74 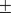 5	1.25 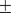 0.21	12.4 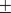 1.5	74 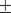 6
Mid brain	2126	1.27 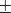 0.25	11.7 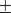 2.2	73 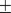 6	1.24 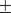 0.26	11.4 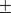 2.4	74 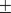 7
Thalamus	1998	1.34 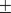 0.15	12.7 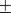 1.1	72 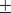 5	1.22 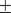 0.28	11.7 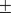 2.4	75 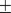 7
Occipital white matter	4608	1.22 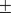 0.36	11.6 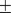 1.9	74 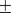 9	1.13 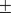 0.34	11.1 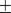 2.2	76 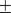 9
Frontal white matter	26450	1.30 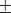 0.37	11.6 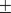 1.9	72 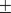 10	1.18 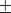 0.39	10.8 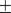 2.4	74 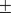 10
Parietal white matter	6706	1.31 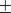 0.34	11.6 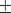 1.7	72 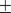 9	1.18 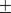 0.38	10.8 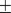 2.3	74 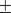 10
Sublobar white matter	3040	1.33 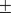 0.40	11.0 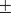 2.7	70 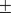 10	1.19 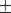 0.46	9.9 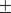 3.3	74 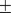 12
White matter	78549	1.35 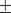 0.34	11.7 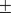 1.7	71 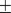 9	1.22 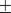 0.37	10.8 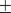 2.3	74 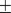 9
Corpus callosum	770	1.31 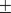 0.53	10.3 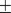 3.4	70 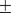 12	1.18 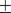 0.53	9.3 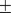 3.6	72 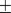 13

The table shows ROI sizes, mean R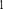, R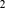, and proton density (PD) values, and standard deviations of 15 pre-defined structures in the Montreal Neurological Institute (MNI) standard brain template. The ROIs were cropped with 2 mm from the standard brain templates. All comparisons between the reference group and the multiple sclerosis group were highly significant, p
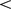
0.001.

**Figure 6:**
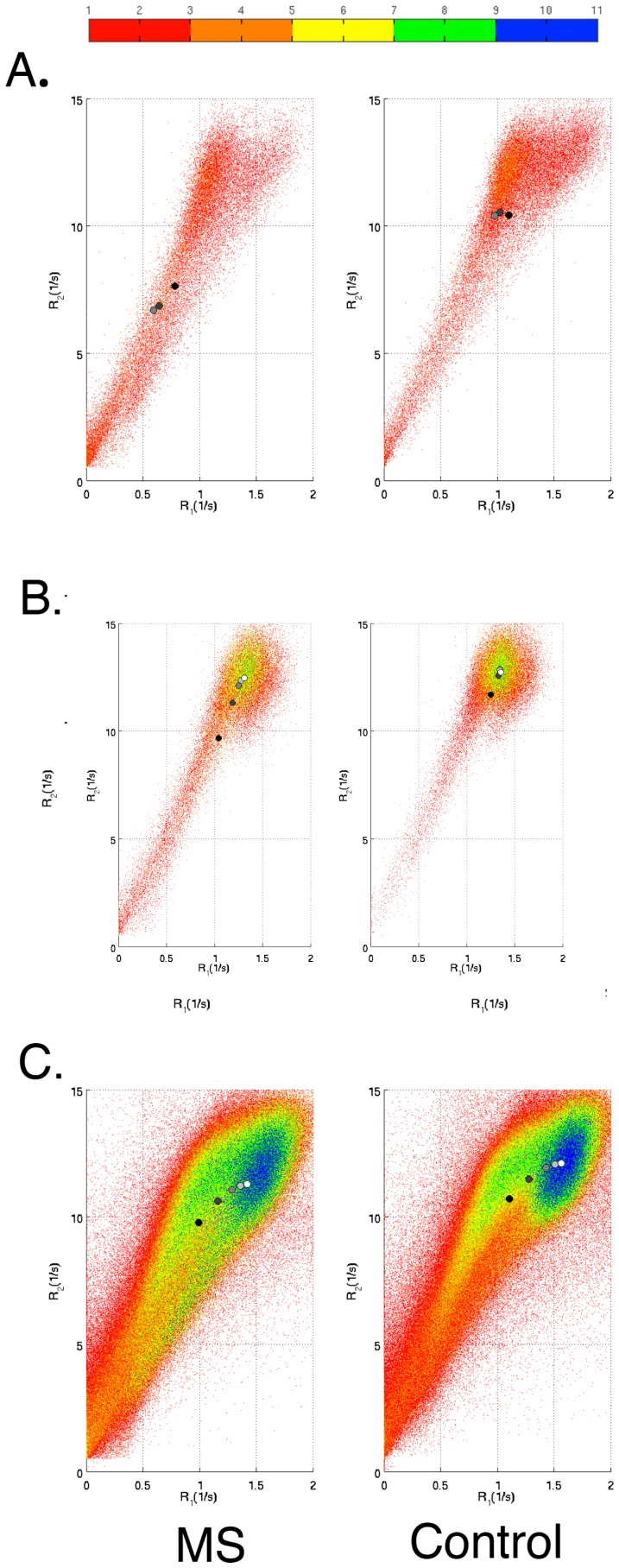
Effect of regions of interest (ROI) size on tissue parameters. The figure shows R
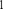
–R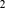 plots in ROIs for the Multiple Sclerosis (MS) and the reference group, respectively. A) Caudate nucleus; B) Thalamus; C) Total white matter. The color bar shows within-ROI distance from the ROI surface. The circles show mean R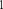 and R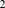 values in 0, 2, 4, 6, and 8 mm reduced ROIs. Black circles represent 0 mm reduction and white circles represent 8 mm reduction.

In Figure 6 the trends regarding changes in 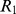
–R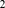 values due to ROI reduction are visualized. The mean 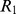
–R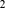 values in each ROI changed towards the 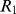
–R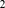 values in the voxels most distant from the ROI surface upon ROI reduction. This feature is clearly seen for the thalamus (Figure 6B) and WM (Figure 6C).

## Discussion

In this study we explored the ability of voxel-based image analysis of qMRI data to assess disease-specific features in groups. We showed that different multi-parametric representations could visualize different properties of brain structure in healthy individuals and MS patients.

### Tissue characterization in groups

The advantage of voxel-based image analysis is the possibility of statistical descriptions of disease-specific features in groups of patients. This means that features that are common in several patients are highlighted whereas the impact of individual patients' focal changes is reduced. Using qMRI, such statistical descriptions typically result in ‘lesion probability’ maps where anatomical structures with the highest probability to find disease-specific lesions are visualized. The statistical treatment of qMRI data also provides a powerful tool to detect diffuse tissue changes that are difficult to distinguish by conventional MRI. Results from the group-level statistical analysis may guide radiologists where in the image to inspect for early signs of disease. However, future research have to validate the method by longitudinal clinical studies in larger study groups.

In this study, we observed distinct aberrations in periventricular white matter, corona radiata, and radiatio optica in MS patients compared to the reference group (Figure 2). Since the images are based on statistical analysis, they show the common pattern across the MS group. Individual lesions that are scattered in white matter and appear at different locations in each patient will be filtered away, and will therefore not show up in the images. If the prevalence of lesions at the same geometrical location is high, however, the average value can be significantly changed compared to the reference group. Therefore we interpret distinct aberrations in periventricular white matter, corona radiata, and radiatio optica to represent MS lesion probability. These locations coincide to a large extent with the lesion probability maps presented in the work by Hasan and co-workers [3].

By inspecting Figure 2, it is clear that MS patients had wide-spread white matter changes especially in central and sub-cortical WM structures. MS patients had reduced R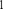 and R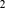, and increased PD compared to the reference group. We interpret these findings to reflect diffuse changes in normal appearing WM. In previous studies, decreased R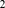 in WM has been related to diffuse myelin or axonal pathology in MS [15], [16]. The qMRI method, applied in the present work, opens up the possibilities to incorporate a myelin model to assess the degree of myelin damage [17]. This approach would enable more precise delineation of the neuropathological basis to the changes in qMRI parameters.

Abnormal relaxation rates and especially increased PD that was observed in the ventricles and cortical sulci of MS patients were probably related to brain atrophy causing enlarged ventricles and wider sulci. Although the brain normalization procedure tries to adjust for individual differences in brain volume, this procedure is not successful in all cases. Calculation of the ventricle fraction showed that the MS patients had significantly larger ventricles than the reference group prior to normalization. By inspecting individual images of MS patients, we observed that several patients had enlarged ventricles also after normalization (see section *Strengths and limitations* for further discussion on this topic).

### Relation to clinical measures

Disorders of the brain are commonly diagnosed based on clinical, laboratory, and neuroimaging data. In MS, typical WM lesions, observed by MRI, are clear signs of disease and provide a solid ground for diagnosis [18]. Thus, conventional MRI provides important information for MS diagnosis and also about the lesion burden in MS. However, the correlation between the number of lesions and the degree of disability in MS is rather weak, and some patients have no visible lesions although they are diagnosed with MS based on clinical information and disease history. This discrepancy between radiological and clinical measures has been termed as the clinico-radiological paradox [14].

In the current study we investigated the possibility to correlate quantitative measures of brain tissue structure and clinical measures on a voxel-by-voxel basis. For this purpose we correlated the R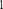, R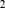, and PD values of each image voxel to the EDSS scores of each MS patient. By this method we showed that the tissue parameter values in frontal WM, especially in and adjacent to the corpus callosum, were correlated to the disease burden, as measured by the EDSS scores. This finding stands in contrast to the observation that WM in the posterior corpus callosum was commonly affected in the MS group independent of disability state. These results suggest that diffuse changes in frontal WM are severe for the individual patient and such information could therefore be indicative for prognosis and treatment strategies. In this context it is important to emphasize that the abnormal tissue parameter values detected by the qMRI method does not directly correspond to focal changes such as MS lesions. This issue is highlighted by the comparison between the present results and those of Kincses and co-workers who correlated MS lesion probability and EDSS [19]. They found that EDSS correlated with MS lesion probability most of all in the posterior periventricular WM.

In this study, we used MS as benchmark sample and EDSS as an example of a commonly used clinical scoring system. It must, however, be noted that EDSS is a rather coarse clinical measure, which especially relates to physical disability (motor dysfunction). The small sample size and the wide range of EDSS (disability scores) in this study, and the big variation of lesion localization in MS patients, must be kept in mind when interpreting the results of this study. Also, the mean MSSS being larger than mean EDSS indicates a proportion of severe cases in our benchmark sample.

Extending the voxel-based qMRI method for assessment of other disorders of the brain than MS, other clinical measures likely would be relevant, for example subjective symptom ratings, cognitive performance scores, or protein and genetic biomarkers, which may reflect significant deviations in other areas of the brain.

### Multi-parametric visualization

In Figure 2, the qMRI parameters are visualized by the conventional geometric representation. Significant differences between two groups (in this case MS patients and healthy individuals) are shown in a data space represented by coronal, axial, and sagittal images. By the geometric representation it is, however, difficult to observe the relations between different qMRI parameters. Alternatively, multi-parametric representations of R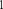, R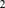, and PD pairs in 2-dimensional graphs provide visualization of different tissue types in segmented clusters. The parametric representation for a single patient has been presented previously by Alfano and co-workers [20]. They used this method to segment normal WM, GM, and CSF for volume calculations of individual subjects' brains. In our work, we extrapolated this method to comparisons of healthy and pathological brain tissue in groups, which to our knowledge have not been presented before. In this way we obtained additional information about disease-specific features.

In our benchmark case, we observed that MS patients had a larger number of voxels in the lower left quadrant in the R
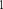
–R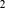 space and in the higher left quadrant in the PD–R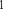 and PD–R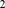 spaces as compared to the reference group of healthy individuals. These representations show differences between MS patients and healthy individuals and highlight disease-specific features in the parameter space. Such multi-parametric representations could eventually be used for assessment of disease progression in individual patients using e.g. pattern recognition or deforming models.

### Tissue properties in regions of interest

Brain normalization and voxel-based statistics of quantitative image data may form a way to automated pathology detection. Hasan and co-workers recently reported an atlas-based approach on multimodal MRI measuring volumetry, diffusimetry, relaxometry and lesion distribution in MS patients [3], [21]. By this approach, they could demonstrate that cerebral pathology in MS is widespread and not limited to MRI visible lesions. Similar results were obtained in the current study, as will be discussed below.

Despite the convincing results, an indiscriminate use of atlas-based ROI analyses could be problematic, because brain normalization is always accompanied by registration errors and atlas-based ROIs are only approximate. Brain normalization is especially complex in patients with a high degree of lesion load and atrophy, which often occurs in MS. Results may also depend on the choice of reference group that is used for preparing the atlas. Yet another problem is the accurate definition of the ROIs: by applying a rather large ROI on a (by normalization) deformed brain will inevitably lead to partial volume effects and hence each ROI will to a certain extent contain information about different tissue types. In our study, we examined this by calculating the distributions of the quantitative measures in each ROI and also in reduced ROI sizes. We found, for example, that R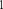 and R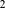 decreased and PD increased in the ventricles upon ROI reduction. Simultaneously, the opposite behavior was observed for regions adjacent to CSF such as the corpus callosum and the pons. These findings indicate that partial volume effects in the ROI analyses were substantial, because reducing the ventricular ROI resulted in increased PD in the ventricles (and decreased R
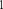
–R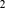) significant of increased CSF fraction in the ventricular ROI. Correspondingly, reducing the corpus callosum and pons ROIs resulted in decreased PD (and increased R
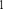
–R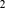) indicating that the reduced ROIs contained a larger fraction of cerebral tissue.

### Quantitative MRI in MS

Several quantitative MRI approaches have been used to assess pathological tissue in MS. The most widely used approaches are diffusion tensor imaging (DTI) [22] and magnetization transfer imaging (MTI) [23]. Both DTI [22], [24], [25] and MTI [26], [27] allow for voxel-based statistical mapping and automated atlas-based analysis [28]. DTI measures and quantifies the diffusion of water molecules parallel or perpendicular to the axonal fibers, for example by calculating the fractional anisotropy (FA), which is a measure of the degree of diffusion directionality. In MS this can provide information about axonal damage and demyelination [22]. A low magnetization transfer ratio (MTR) is an indicator of reduced interaction between protons in free water and protons bound to macromolecules. In MS, demyelination is the most probable cause of an MTR reduction [23]. Quantitative MRI, which was utilized in the present study, provides information about the tissue parameters that give rise to the contrast in conventional MRI. PD gives information about the water content in tissue, and it is therefore sensitive to both edema and atrophy when brain tissue is replaced by CSF. R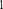 and R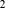 are the longitudinal and transversal relaxation rates, respectively, which magnitudes are affected by the tissue structure and the influence of magnetic exchange, especially with fast-relaxing myelin water. In this work only the main R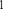 and R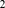 components were measured. A potential extension of the technique might incorporate multi-exponential relaxation behavior as well.

### Strengths and limitations

The strength of the qMRI method used in this study is the relatively short scanning time and the simultaneous acquisition of all three qMRI parameters in a single experiment. Depending on resolution the sequence requires 5 to 8 minutes, which enables readily introduction of qMRI sequences into conventional clinical routines. Additionally, by simultaneous measurements of all quantitative parameters the often problematic procedure of image registration in single subject imaging is avoided. Since qMRI is independent of scanner settings, such as echo time (TE) and repetition time (TR), and scanner imperfections, such as coil sensitivity of B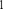 inhomogeneity, it is mapped on an absolute scale. This should improve the group statistics in comparison to conventional imaging, which is acquired on a relative scale. Moreover, qMRI forms a robust input for automatic brain segmentation procedures, which could provide complementary information about differences in brain volume [29], [30].

Perhaps the main limitations of the present study are the rather large image voxel sizes and the non-isotropic image acquisition. These limitations have large impact on normalization quality. As discussed above, the normalization procedure failed to normalize ventricle sizes in MS patients. Non-optimized normalization was also observed in other cortical areas, especially in the occipital lobe, in some subjects. Various degrees of brain atrophy of the subjects generally constitute a problem for normalization procedures due to the complex geometry of the brain and the small, partial-volume areas filled with CSF. The relatively low resolution causes a relatively large partial volume effect, as demonstrated in Tables 1 and 2. Using smaller, isotropic voxels during image acquisition would substantially improve normalization and it would also allow refined normalization procedures for example using the method of nonlinear registration by local optimization as implemented in the DARTEL tool [31].

## Conclusions

In the present study, we showed that multi-parametric representations of qMRI data could highlight deviations in cerebral structure in patients with brain disorders. The proposed method could visualize both high-probability focal anomalies and diffuse tissue changes. Thus, results from voxel-based statistical analysis, as exemplified in the present work, may guide radiologists where in the image to inspect for early signs of disease. Additionally, the fast imaging protocol facilitates the introduction of the qMRI method into clinical practice.

## Materials and Methods

### Ethics statement

The study was approved by the Regional Ethical Review Board in Linköping (Dnr. M88-07) and written informed consent was obtained from all participants.

### Subjects

For generating the reference tissue maps, 19 healthy controls were recruited to the study (5 were males and 14 were females). In addition, 19 patients that were diagnosed with clinically definite MS were included. Five patients were male and 14 females. The mean age of the MS patients was 47.7
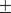
11.9 years. The mean age difference between the healthy controls and the MS patients was 1.1
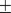
2.6 years. All 19 MS patients fulfilled the Barkhof-Tintorée criteria of MS diagnosis [32] in prior MRI examinations. The mean duration of disease was 15.1
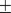
10.2 years. The disability status of the MS patients was rated with EDSS [33] and the Multiple Sclerosis Severity Score (MSSS) [34]: mean EDSS = 3.7 (range = 1–8.5), mean MSSS = 4.2 (range = 0.5–9.6).

### Scanning protocol

For qMRI the QRAPMASTER sequence [12] was used. QRAPMASTER is a multi spin-echo saturation recovery sequence with 4 saturation delays and 5 echoes. The saturation delays were at 100, 400, 1380, and 2860 ms with a TR of 2950 ms. TE was set to 14, 28, 42, 56, and 70 ms. Hence each acquisition resulted in a matrix of 4
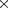
5 = 20 images per slice with different effects of T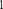 and T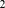 relaxation on the image signal intensity. The in-plane resolution was 1
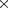
1 mm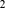 over a field of view (FOV) of 210 mm. Thirty axial slices of 4 mm thickness (no gap) were collected in a scan time of 8∶21 minutes. The MR-scanner was an Achieva 1.5 T (Philips Healthcare, Best, The Netherlands).

### Image post-processing

Image data were analyzed with the SyMRI 7.0 software (SyntheticMR AB, Linköping, Sweden) to retrieve the R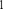 = 1/T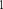, R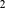 = 1/T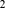, and PD maps as described in Warntjes *et al.* [12]. Furthermore a stack of T2-weighted images was recreated based on the same dataset using the approach of synthetic MRI [35], [36]. The R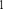, R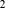, and PD maps were normalized to a standard stereotactic space in Montreal Neurological Institute (MNI) co-ordinates using SPM8 (Wellcome Department of Imaging Neuroscience, University College, London, UK), as described in previous works by us [13], [37]. Before normalization, the synthetic T2-weighted images were smoothed with an 8 mm Gaussian kernel to reduce the individual anatomical details, and thereafter used as source images to calculate the transformation matrices between individual images and the template. The normalization to the MNI space was done by a 12-parameter (translation, rotation, shear, zoom) affine registration followed by nonlinear deformations, defined by a linear combination of three-dimensional discrete cosine basis functions. The resulting transformation matrices were then applied to the WM, GM, and CSF maps. The resulting maps were re-gridded to 2×2×2 mm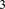 voxel size to obtain an isotropic dataset. Voxel-wise differences in R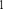, R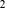, and PD between MS patients and healthy controls were estimated by two-sample t-tests controlling for age. For the MS group, all voxels of the parameter maps (R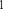, R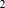, and PD) were correlated to EDSS with age as a nuisance variable.

Brain segmentation was performed on the intracranial volume, brain volume, and cerebrospinal fluid volume, using the automatic segmentation algorithm of SyMRI 7.0. In summary, the tissue classes white matter, grey matter and CSF are determined as a combination of specified R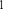, R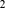 and PD value ranges. Partial volume is estimated for intermediate R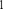, R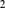 and PD values. The brain volume is calculated as a contiguous volume containing white and grey matter. The intracranial volume is calculated a contiguous volume containing brain tissue and CSF. The ventricular volume was obtained by manually selecting the lateral ventricles in the provided cerebrospinal fluid map. The brain parenchymal fraction was calculated as the brain volume divided by the intracranial volume. The ventricular fraction was calculated as the ventricular volume divided by the intracranial volume.

### Region of interest analysis

The Wake Forrest University (WFU) PickAtlas [28] defined in the standard MNI space was used to define ROIs in the cingulate cortex, insula, putamen, thalamus, pons, and the midbrain. We also made ROIs representing whole brain grey matter (GM) and regions of frontal, parietal, occipital, and sub-lobar white matter (WM) as well as ROIs representing the corpus callosum and the lateral ventricle.

Statistical significance of differences in R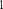, R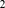, and PD between the reference group and MS patients, as well as the EDSS correlation in the MS group, was assessed by small volume corrections in brain parenchyma ROIs. Results were considered significant if p
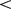
0.05, family wise error (FWE) rate corrected for multiple comparisons regarding the number of voxels in each ROI and the number of ROIs used in each test.

Differences in mean R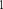, R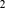 and PD were evaluated using a mixed linear model in R [38] using the lme4 [39] package. Group was used as a fixed factor and each subject and voxel were treated as random samples.

Since it was expected that these standardized ROIs would involve substantial partial volume effects, we also did the ROI analyses in reduced ROIs. We reduced the ROI volumes by eroding all voxels that had a distance of 2 mm or lower to the edge of the ROI. The ROI reduction was performed in steps of 2 mm and we calculated the mean R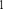, R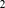, and PD in each reduced ROI. The reduction was performed on the data after normalization to standard MNI isotropic 2 mm resolution.

## References

[1] Tofts P, editor (2003) Quantitative MRI of the brain. Wiley.

[2] Kumar R, Delshad S, Woo MA, Macey PM, Harper RM (2012) Age-related regional brain t2-relaxation changes in healthy adults. J Magn Res Imag 35: 300–308. 10.1002/jmri.22831 21987489

[3] Hasan KM, Walimuni IS, Abid H, Wolinsky JS, Narayana PA (2012) Multimodal quantitative MRI investigation of brain tissue neurodegeneration in multiple sclerosis. J Magn Res Imag 35: 1300–1311. 10.1002/jmri.23539 PMC3330152 22241681

[4] Draganski B, Ashburner J, Hutton C, Kherif F, Frackowiak RSJ, et al (2011) Regional specificity of MRI contrast parameter changes in normal ageing revealed by voxel-based quantification (VBQ). NeuroImage 55: 1423–1434. 21277375 10.1016/j.neuroimage.2011.01.052 PMC3093621

[5] Neema M, Stankiewicz J, Arora A, Dandamudi VS, Batt CE, et al (2007) T1- and T2-based MRI measures of diffuse gray matter and white matter damage in patients with multiple sclerosis. J Neuroimag 17: 16S–21S. 10.1111/j.1552-6569.2007.00131.x 17425729

[6] Neeb H, Zilles K, Shah NJ (2006) A new method for fast quantitative mapping of absolute water content in vivo. NeuroImage 31: 1156–1168. 16650780 10.1016/j.neuroimage.2005.12.063

[7] Oh J, Cha S, Aiken AH, Han ET, Crane JC, et al (2005) Quantitative apparent diffusion coefficients and T2 relaxation times in characterizing contrast enhancing tumors and regions of peritumoral edema. J Magn Res Imag 21: 701–708. 10.1002/jmri.20335 15906339

[8] Deoni SCL, M PT, K RB (2005) High-resolution T1 and T2 mapping of the brain in a clinically acceptable time with DESPOT1 and DESPOT2. Magn Res Med 53: 237–241. 10.1002/mrm.20314 15690526

[9] Deichmann R (2005) Fast high-resolution T1 mapping of the human brain. Magn Res Med 54: 20–27. 10.1002/mrm.20552 15968665

[10] Larsson HB, Frederiksen J, Petersen J, Nordenbo A, Zeeberg I, et al (1989) Assessment of demyelination, edema, and gliosis by in-vivo determination of T1 and T2 in the brain of patients with acute attack of multiple sclerosis. J Magn Reson Med 11: 337–338. 10.1002/mrm.1910110308 2779421

[11] Warntjes J, Dahlqvist O, Lundberg P (2007) A novel method for rapid, simultaneous *t* _1_,*t* _2_ ^*^ and proton density quantification. Magn Res Med 57: 528–537. 10.1002/mrm.21165 17326183

[12] Warntjes J, Dahlqvist Leinhard O, West J, Lundberg P (2008) Rapid magnetic resonance quantification on the brain: Optimization for clinical usage. Magn Res Med 60: 320–329. 10.1002/mrm.21635 18666127

[13] Warntjes J, Engström M, Tisell A, Lundberg P (2012) Brain characterization using normalized quantitative magnetic resonance imaging. Plos One 8: e70864. 10.1371/journal.pone.0070864 PMC3733841 23940653

[14] Barkhof F (2002) The clinico-radiological paradox in multiple sclerosis revisited. Curr Opin Neurol 15: 239–245. 12045719 10.1097/00019052-200206000-00003

[15] Neema M, Goldberg-Zimring D, Guss ZD, Healy BC, Guttmann CRG, et al (2009) 3 T MRI relaxometry detects T2 prolongation in the cerebral normal-appearing white matter in multiple sclerosis. NeuroImage 46: 633–641. 19281850 10.1016/j.neuroimage.2009.03.001 PMC2974316

[16] Whittall KP, MacKay AL, Li DKB, Vavasour IM, Jones CK, et al (2002) Normal-appearing white matter in multiple sclerosis has heterogeneous, diffusely prolonged T2. Magn Res Med 47: 403–408. 10.1002/mrm.10076 11810687

[17] Levesque R, Pike GB (2009) Characterizing healthy and diseased white matter using quantitative magnetization transfer and multicomponent T_2_ relaxometry: A unified view via a four-pool model. Mag Reson Med 62: 1487–1496. 10.1002/mrm.22131 19859946

[18] Filippi M, Agosta F (2010) Imaging biomarkers in multiple sclerosis. J Magn Res Imag 31: 770–788. 10.1002/jmri.22102 20373420

[19] Kincses ZT, Ropele S, Jenkinson M, Khalil M, Petrovic K, et al (2010) Lesion probability mapping to explain clinical deficits and cognitive performance in multiple sclerosis. Mult Scler J 17: 681–689. 10.1177/1352458510391342 21177325

[20] Alfano B, Brunetti A, Covelli EM, Quarantelli M, Panico MR, et al (1997) Unsupervised, automated segmentation of the normal brain using a multispectral relaxometric magnetic resonance approach. Magn Res Med 37: 84–93. 10.1002/mrm.1910370113 8978636

[21] Hasan KM, Walimuni IS, Abid H, Datta S, Wolinsky JS, et al (2012) Human brain atlas-based multimodal MRI analysis of volumetry, diffusimetry, relaxometry and lesion distribution in multiple sclerosis patients and healthy adult controls: Implications for understanding the pathogenesis of multiple sclerosis and consolidation of quantitative MRI results in MS. J Neurol Sci 313: 99–109. 21978603 10.1016/j.jns.2011.09.015 PMC3254796

[22] Roosendaal S, Geurts J, Vrenken H, Hulst H, Cover K, et al (2009) Regional DTI differences in multiple sclerosis patients. NeuroImage 44: 1397–1403. 19027076 10.1016/j.neuroimage.2008.10.026

[23] Filippi M, Rovaris M (2000) Magnetisation transfer imaging in multiple sclerosis. J Neuro Virol 6: 115–120. 10871798

[24] Ceccarelli A, MA R, Pagani E, Ghezzi A, Capra R, et al (2008) The topographical distribution of tissue injury in benign MS: A 3T multiparametric MRI study. NeuroImage 39: 1499–1509. 18155611 10.1016/j.neuroimage.2007.11.002

[25] Bodini B, Khaleeli Z, Cercignani M, Miller D, Thompson A, et al (2009) Exploring the relationship between white matter and gray matter damage in early primary progressive multiple sclerosis: An in vivo study with TBSS and VBM. Hum Brain Map 30: 2852–2861. 10.1002/hbm.20713 PMC6871131 19172648

[26] Audoin B, Ranjeva JP, Van Au Duong M, Ibarrola D, Malikova I, et al (2004) Voxel-based analysis of MTR images: A method to locate gray matter abnormalities in patients at the earliest stage of multiple sclerosis. J Magn Res Imag 20: 765–771. 10.1002/jmri.20178 15503338

[27] Jure L, Zaaraoui W, Rousseau C, Reuter F, Rico A, et al (2010) Individual voxel-based analysis of brain magnetization transfer maps shows great variability of gray matter injury in the first stage of multiple sclerosis. J Magn Res Imag 32: 424–428. 10.1002/jmri.22265 20677272

[28] Maldjian JA, Laurienti PJ, Kraft RA, Burdette JH (2003) An automated method for neuroanatomic and cytoarchitectonic atlas-based interrogation of fMRI data sets. NeuroImage 19: 1233–1239. 12880848 10.1016/s1053-8119(03)00169-1

[29] Vågberg M, Lindqvist T, Warntjes JBM, Sundström P, Birgander R, et al (2013) Automated determination of brain parenchymal fraction in multiple sclerosis. Am J Neuroradiol 34: 498–504. 22976234 10.3174/ajnr.A3262 PMC7964911

[30] Ambarki K, Lindqvist T, Wåhlin A, Petterson E, Warntjes M, et al (2012) Evaluation of automatic measurement of the intracranial volume based on quantitative MR imaging. Am J Neuroradiol 33: 1951–1956. 22555574 10.3174/ajnr.A3067 PMC7964602

[31] Ashburner J (2007) A fast diffeomorphic image acquisition algorithm. NeuroImage 38: 95–113. 17761438 10.1016/j.neuroimage.2007.07.007

[32] McDonald WI, Compston A, Edan G, Goodkin D, Hartung HP, et al (2001) Recommended diagnostic criteria for multiple sclerosis: guidelines from the international panel on the diagnosis of multiple sclerosis. Ann Neurol 50: 121–127. 11456302 10.1002/ana.1032

[33] Kurtzke J (1983) Rating neurologic impairment in multiple sclerosis: An expanded disability status scale (EDSS). Neurology 33: 1444–1452. 6685237 10.1212/wnl.33.11.1444

[34] Roxburgh R, Seaman SR, Masterman T, Hensiek AE, Sawcer SJ, et al (2005) Multiple sclerosis severity score. using disability and disease duration to rate disease severity. Neurology 64: 1144–1151. 15824338 10.1212/01.WNL.0000156155.19270.F8

[35] Bobman S, Riederer S, Lee J, Suddarth S, Wang B, et al (1985) Cerebral magnetic resonance image synthesis. Am J Neuro Rad 6: 265–269. PMC8332877 2984911

[36] Riederer S, Lee J, Farzeneh F, Wang H, Wright R (1986) Magnetic resonance image synthesis: Clinical implementation. Acta Radiol 369: 466–468. 2980529

[37] West J, Blystad I, Engström M, Warntjes JBM, Lundberg P (2013) Application of quantitative MRI for brain tissue segmentation at 1.5 T and 3.0 T field strengths. Plos One 8: e74795. 24066153 10.1371/journal.pone.0074795 PMC3774721

[38] Team RC (2013) R: A language and environment for statistical computing. R Foundation for Statistical Computing, Vienna, Austria. Http://www.R-project.org.

[39] Bates D, Maechler M, Bolker B, Walker S (2013) lme4: Linear mixed-effects models using Eigen and S4, R package version 1.0-4 edition. Http://CRAN.R-project.org/package=lme4.

